# Cajal, Retzius, and Cajal–Retzius cells

**DOI:** 10.3389/fnana.2014.00048

**Published:** 2014-06-17

**Authors:** Verónica Martínez-Cerdeño, Stephen C. Noctor

**Affiliations:** ^1^Institute for Pediatric Regenerative Medicine, University of California at DavisSacramento, CA, USA; ^2^Medical Pathology and Laboratory Medicine, University of California at DavisSacramento, CA, USA; ^3^MIND Institute, University of California at DavisSacramento, CA, USA; ^4^Department of Psychiatry and Behavioral Sciences, University of California at DavisSacramento, CA, USA

**Keywords:** Ramón y Cajal, Cajal–Retzius cells, marginal zone, layer I, horizontal cells, short axon cells, Reelin

## Abstract

The marginal zone (MZ) of the prenatal cerebral cortex plays a crucial role in cellular migration and laminar patterning in the developing neocortex and its equivalent in the adult brain – layer I, participates in cortical circuitry integration within the adult neocortex. The MZ/layer I, which has also been called the plexiform layer and cell-poor zone of Meynert, among others, is home to several cell populations including glia, neurons, and Cajal–Retzius (CR) cells. Cajal once said that the MZ is one of the oldest formations in the phylogenetic series, and that the characteristics of layer I in human are similar in all vertebrates except fish (Ramon y Cajal, 1899). Despite the presence of CR cells in the MZ/layer I of all developing and adult vertebrate brains, and more than one hundred years of research, the phenotype and function of layer I cells have still not been clearly defined. Recent technological advances have yielded significant progress in functional and developmental studies, but much remains to be understood about neurons in MZ/layer I. Since the time of Retzius and Cajal, and continuing with modern era research from the likes of Marín-Padilla, the study of CR cells has been based on their morphological characteristics in Golgi staining. However, since Cajal’s initial description, the term “CR cell” has been applied differently and now is often used to indicate reelin (Reln)-positive cells in MZ/layer I. Here we review the history of work by Cajal, Retzius, and others pertaining to CR cells. We will establish a link between original descriptions of CR cell morphology by Cajal, Retzius, and others, and current understandings of the cell populations that reside in MZ/layer I based on the use of cellular markers. We propose to use the term “CR cell” for the class of neurons that express Reln in the MZ/layer I in both prenatal, developing and adult cerebral cortex.

## POSTNATAL MAMMALIAN LAYER I BY CAJAL

Neurons in layer I were first described by Ramon y Cajal (1890). After several years of work using a variety of staining protocols including the Golgi method, Cajal concluded that there were two types of cells in layer I of the postnatal mammalian cerebral cortex: large horizontally oriented neurons, and neurons with a short axon [also called “Golgi corpuscle cells” by Ramon y Cajal (1897)]. Cajal also noted that other cellular structures were present in layer I including terminal dendritic bouquets, ascending axonal arborizations, and glial cells. This review will focus on neurons that have their soma within layer I: Cajal’s horizontal cells, and cells with a short axon. We will contrast the description of these cells by Cajal and his colleagues more than one hundred years ago with current information.

In 1890, Cajal described a new type of large fusiform neuron in layer I of neonatal rabbit cerebral cortex that had a horizontal orientation. He collected additional data from the neonatal cerebral cortex of cat, rabbit, rat, and dog to show that in each species these cells were scarce and positioned at different levels in layer I, particularly deeper portions of layer I and superficial portions of layer II. These horizontal cells possessed processes that were parallel to the pia and from which originated thin branches, one of which was an axon. The axonal process of these cells was very long and thick with ascending collaterals that extended at right or obtuse angles to terminate exclusively within layer I (Ramon y Cajal, 1895).

In 1895, Cajal described a second distinct population of layer I neurons that possessed a short axon. These cells were distributed equally at different levels within layer I – superficial, intermediate and deeper portions – in neonatal rabbit and rat. The short axon cells exhibited several different morphological shapes and sizes, but tended to be larger in deeper portions of layer I, and gave rise to a large number of dendrites extending from the perikarion in all directions, but primarily toward the pial surface (Ramon y Cajal, 1895). The dendrites of short axon cells were usually very short, branched near the soma, and did not exit layer I (Ramon y Cajal, 1899). Based on morphology, he described at least two subtypes of short axon cells in mammals and at least four principle subtypes in human. He used the terms common type or medium-sized, large type, small type, and neurogliaform type to distinguish these cell subtypes. He determined that the medium-sized cells were the most abundant of the short axon cells in layer I and were positioned within intermediate and deep portions of layer I. The medium-sized cells were characterized by ascending dendrites and an axon that coursed parallel to the pial surface of the cortex. The large type possessed long, thick dendrites that descended into layers II and III, and a thick axon that ran parallel to the pia within layer I. The small cell type had small oval or piriform soma and a thin axon that ramified near the cell body. Some of the small cells were positioned very close to the pia. Finally, Ramon y Cajal (1895) described the neurogliaform type as dwarf cells in the deepest portion of layer I that elaborated very complex and dense terminal arborizations.

## PRENATAL HUMAN MARGINAL ZONE BY RETZIUS

In 1891, one year after Cajal described horizontal cells in layer I of the postnatal rabbit cortex, Retzius described cells located within the outer layer of the cerebral cortex in human fetuses at six to eight months of gestation. In his writings, Retzius commented that layer I cells had previously been noted by many other neuroanatomists using Golgi and other techniques, including Meynert, Henle, Schwalbe, Krause, Ranvier, Toldt, Kahler, and Obersteiner. However, due to the difficulty of impregnating these cells with the Golgi technique, and since some of these researchers thought that Golgi-labeled structures in layer I were artifacts, and other thought that they were glial cells, these layer I cells were not described in any detail. Therefore, it was Cajal and then Retzius who first dedicated time to study these complex layer I cells (Retzius, 1893).

Retzius first studied the MZ in the developing cerebral cortex using Golgi stained material from late gestation fetal human, and later in prenatal mouse, rat, cat, and dog. He noted that it was difficult to obtain good results using the Golgi method, that he was only able to stain a small proportion of MZ cells in third-trimester human cortex, and that he was not able to stain these cells in fetal human cortex younger than 6 months of gestation, nor in the brains from young children. His first opinion was that the cells he stained within the human MZ were very different both in form and orientation from the cells described one year earlier by Cajal in postnatal rabbit. Retzius initially thought that these cells were glial cells, but later agreed with Cajal that they were neurons, and also concluded that the human cells shared much in common and were potentially related to those described by Cajal. He initially referred to these cells as carrots cells since its body resembled a carrot, but later suggested the name “Cajal cells.” Therefore, it was Retzius who first used the term “Cajal cells” for cells of layer I in postnatal animals and fetal human. He described how the “Cajal cells” in humans exhibited soma with different morphological shapes and had cellular processes that were more numerous and elaborate than in other mammals. It is important to note that the cells first described by Cajal had a precisely positioned horizontal soma, but this was not the case for the cells described by Retzius (1893). Furthermore, if we were to stain the cells described by Cajal and by Retzius with somatic markers available today, such as Reln, they may not appear to be related cell types based on morphology.

**FIGURE 1:**
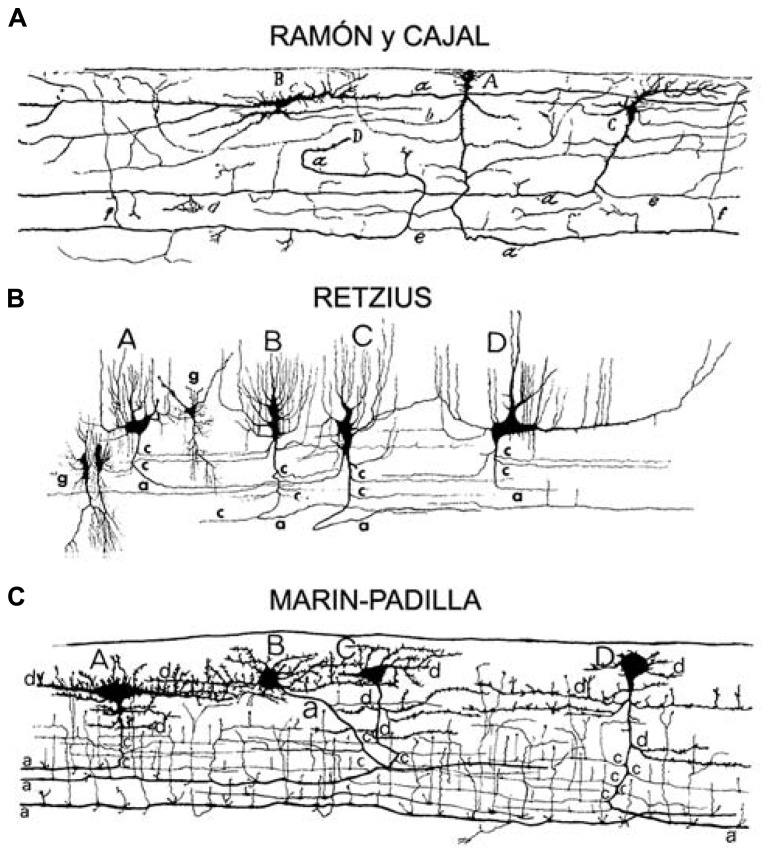
**Adapted from** Marin-Padilla (1990). Original drawings of Cajal–Retzius cells from the motor cortex of a **(A)** 2-month-old infant (Ramon y Cajal, 1911), **(B)** a 26-week-old fetus (Retzius, 1894), **(C)** a newborn (Marin-Padilla and Marin-Padilla, 1982), sagittal perspective of layer I. Pyriform or triangular (Cajal A; Retzius A,B,C; Marin-Padilla C,D), horizontal monopolar or bipolar (Cajal B; Retzius D; Marin-Padilla A), and stellate like (Marin-Padilla B) cell types.

Retzius described human fetal Cajal cells as having round, oval, carrot-like, polygonal, or unevenly shaped soma of different sizes positioned at different levels within layer I, in some cases displaying a parallel orientation and in other cases a perpendicular orientation with respect to the pia (see **Figure 1A**). Retzius showed that these cells had several processes that emanated in various directions from the soma: superficially toward the pial surface, deep into the underlying cortex, or horizontally via small thick processes that often lead toward the surface. They had a total of 6–8 horizontal branches, each elaborating multiple processes extending to the surface and forming a characteristic mesh pattern (Retzius, 1893; Koelliker, 1896).

In a later publication, Cajal again described his two types of layer I cells – the horizontal and short axon cells – and also referred to a third type as “special cells of the cortex named by Retzius as Cajal cells.” This emphasizes that, at that point in history, Ramon y Cajal (1897) did not think that his horizontal cells and the layer I cells described by Retzius were homologous. Soon after Veratti proposed the idea of including the “Cajal cells” described by Retzius within Cajal’s horizontal cell category. Cajal came to accept this proposal, saying “with time I feel less repugnancy toward this idea” (Ramon y Cajal, 1897). In his later publications, Cajal referred to his horizontal cells and to the “Cajal cells” that were described by Retzius as one and the same.

## CAJAL–RETZIUS CELLS BY KOELLIKER

While Retzius was not able to label Cajal cells with the Golgi method in cortical tissue obtained from children, Koelliker (1896) used Golgi and clearly detected such cells in cortical tissue from both children and adults and agreed with Retzius’ descriptions. Koelliker (1896) thought it difficult to discern the function of Cajal cells (in postnatal animals) and Retzius cells (in prenatal human). His opinion was that both cell types were to be kept separate and for this purpose he named them differently. He wrote that Cajal cells were much simpler and that there were fewer Cajal cells than Retzius cells in the MZ/layer I. The characteristic features of Cajal cells were so similar to neurons that he assumed they were indeed nerve cells. In contrast, he believed that the Retzius cells were glial by nature, and he suggested that they were “special glial transitory” cells. He stated that if Retzius cells were nerve cells, he could not explain the function of the multiple extensions ending at the surface, and the function/meaning of the entire structure would be in question. He reasoned that all of the superficial extensions of the Retzius cells ended in the upper surface glial layer of the brain, which is located above the stratum zonale that does not contain any nerve fibers (Koelliker, 1896). Nonetheless, some researchers have suggested that it was Koelliker who coined the term CR cells to describe the layer I cells described by Cajal in neonatal brain, and by Retzius in fetal brain (Huntley and Jones, 1990).

## PRENATAL MARGINAL ZONE AND POSTNATAL LAYER I IN HUMAN BY CAJAL

Cajal followed his discoveries and those of Retzius by carefully studying the morphological similarities and differences between horizontal neurons in fetal MZ and neonatal and adult layer I in human. He concluded that horizontal cells were more abundant and larger in human. Cajal used the Golgi method to examine layer I cells in tissue obtained from seven- to nine-month human fetuses, and reported that horizontal cells were distributed through the entire thickness of the MZ, but were more numerous in close proximity to the pia (Ramon y Cajal, 1899). Agreeing with the descriptions by Retzius, he reported that the morphology of fetal horizontal cells varied, and included cells with fusiform, triangular, stellar, and piriform morphologies. The most characteristic features of these cells were horizontal processes that branched, yielding an extraordinary number of long, varicose horizontal fibers from which sprung at right angles innumerable ascending processes that terminated in rounded knobs near the surface of the cortex. After giving rise to a large number of ascending processes, the horizontal processes became thinner and finally subdivided into terminal branches that extended under the pia or in the most superficial region of the MZ (Ramon y Cajal, 1899). Cajal compared the data he obtained from humans with that obtained from rabbit, cat and other animal species. He concluded that in non-human mammals, fetal horizontal cells give rise to a comparatively smaller number of horizontal branches and that these extended shorter distances than those in human. Cajal compared the MZ in several cortical areas and came to the conclusion that it was populated by the same elements across cortical areas. The only potential difference he noted was that in some areas, for example motor cortex, the MZ was thicker than in others, and Ramon y Cajal (1899) proposed that this resulted from an increased number of pyramidal neuron dendritic terminations and horizontal cells. Cajal also compared the morphological features of cortical CR cells to those in the MZ of cerebellum, hippocampus and dentate gyrus and found them to be similar (Ramon y Cajal, 1899).

**FIGURE 2:**
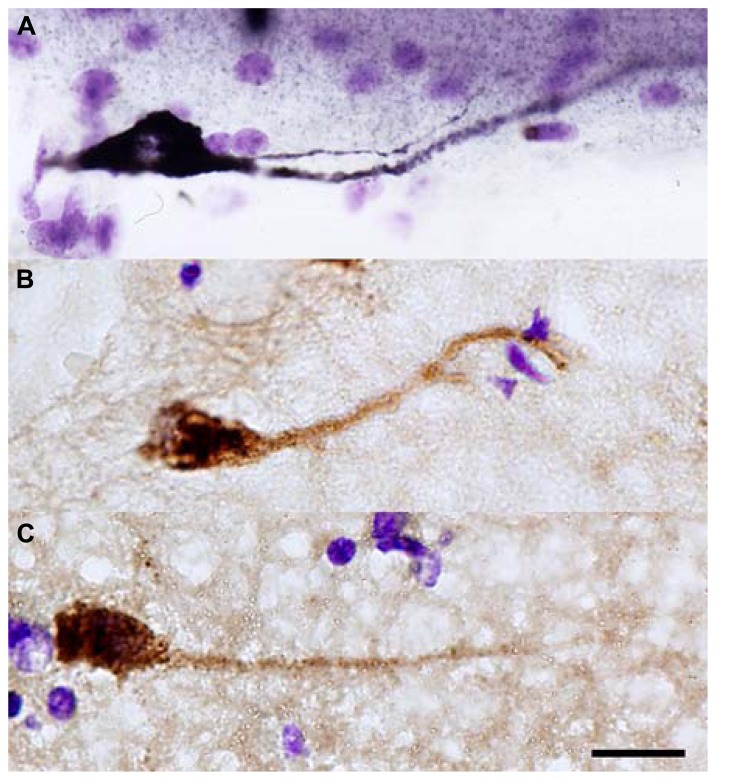
**Reln+ horizontal cells in the temporal cortex of adult macaque (A) and human (B,C)**. Scale bar: 10 μm.

After examining neonatal layer I in tissue obtained from 15- to 30-day-old human brains, Cajal reported that most of the ascending branches described by Retzius in the fetal brain were no longer present after birth, and that the remaining fibers changed directions and ramified within layer I (see **Figure 1**). He determined that layer I cell bodies were smaller in the neonatal brain, and that horizontal projections were thinner in diameter but notably longer when compared to fetal cells (Ramon y Cajal, 1899). These long horizontal branches formed a system of parallel fibers that retained their original orientation. They had a thick myelinated axon that ran parallel to the pial surface for long distances, and issued collateral processes that ramified around the soma of short axon cells in layer I (Ramon y Cajal, 1895). Basing his work on Retzius’ description of horizontal cells in the human fetus, Cajal identified three main morphologies of adult CR cells, unipolar or marginal cells, bipolar cells, and stellate or triangular cells. Cajal was not able to stain adult layer I cells with Golgi but used other techniques, including methylene blue, to describe the presence of short axon cells in adult layer I (“cilindro-eje” cells in Spanish). Cells exhibiting a morphology equivalent to Cajal’s large horizontal cells have since been reported in vertebrates including crocodiles, lizards, non-primate mammals, non-human primates, and human (see **Figure 2**; Ramon y Cajal, 1896; Bernier et al., 1999; Goffinet et al., 1999; Perez-Garcia et al., 2001; Martinez-Cerdeno and Clasca, 2002; Martinez-Cerdeno et al., 2002, 2003; Tissir et al., 2003; Molnar et al., 2006; Ramos-Moreno et al., 2006; Meyer, 2010).

Cajal hypothesized that the function of layer I cells was probably to establish connections between the terminal arborizations of Martinotti cell axons and/or association fibers coming from the white matter, with the terminal branches of the pyramidal neuron apical dendrites. He further proposed that the function of the large horizontal cells was to establish connections between layer I elements that were separated by considerable distances, while short axon cells performed the same function for elements separated by short or moderate distances (Ramon y Cajal, 1899).

## ADULT HUMAN LAYER I BY MARÍN-PADILLA

Neither Retzius nor Cajal were able to Golgi-stain layer I cells in the adult cortex. However, one century later Marín-Padilla successfully used the Golgi method to perform detailed characterizations of cells within layer I of human neocortex (Marin-Padilla and Marin-Padilla, 1982). Marín-Padilla described the presence of cells in layer I of infants (eight-month-old), children (eight-year-old), young adults (16-year-old), and adult (26-year-old; Marin-Padilla, 1990; see **Figure 1B**). Until Marín-Padilla demonstrated the presence of both horizontal and short axons cells in adult layer I, the concept that these cells do not exist in the adult human brain had become widely accepted. The idea that CR cells are not present in the adult may have been initially proposed by Connel (1941, 1947, 1951). This hypothesis was supported by work that showed degenerating CR cells in the MZ of both rodents and humans (Derer and Derer, 1990; Meyer and Gonzalez-Hernandez, 1993; Super et al., 1998; Zecevic and Rakic, 2001; Abraham and Meyer, 2003; Tissir et al., 2009). But, recent work has shown that layer I horizontal neurons that express Reln (CR cells) persist in the adult layer I (see **Figure 2**; Pesold et al., 1999; Martinez-Cerdeno and Clasca, 2002; Martinez-Cerdeno et al., 2002, 2003; Chowdhury et al., 2010). Several factors may have contributed to the concept that CR cells were absent from layer I in adult human neocortex. First, the progressive postnatal expansion of the cerebral cortex dilutes the CR cell population within layer I (Marin-Padilla, 1990). Marin-Padilla studied adult CR cells in detail and concluded that the cortical expansion that occurs during postnatal development impacts the location of CR cell soma and principal dendrites, but does not alter the distribution of the long horizontal axonal processes. As a result, CR cell bodies are only found in specific locations, while the axonal processes are distributed throughout layer I in the entire cerebral cortex (Marin-Padilla, 1990). Furthermore, Marin-Padilla demonstrated that the axonal processes in layer I of any given cortical area represent those of all the CR cells in that area. Therefore, CR cell numbers are low in some cortical regions and the study of many consecutive sections is required to encounter CR cell bodies and principle dendrites. Despite the apparent change in layer I cell density, Marin-Padilla (1990) emphasized that CR cells persist in the adult cerebral cortex with essentially unchanged morphological properties, especially of axonal processes. Second, additional cell populations are present in high numbers in the MZ of the prenatal and developing brain, but are absent or reduced in the adult, contributing to the progressive cell loss in the MZ/layer I. Many cortical interneurons migrate through the MZ en route from the ganglionic eminences to the dorsal neocortex, but leave the MZ to enter the underlying cortical plate (Kriegstein and Noctor, 2004). Cortical microglia are also concentrated in the pial meninges and MZ during development, but achieve and even distribution throughout cortical tissue in the mature brain (Cunningham et al., 2013). Both migrating cortical interneurons and microglia can present with horizontal profiles in the MZ, temporarily adding to the overall population of horizontally oriented cells in the MZ/layer I during development.

## MODERN ERA CAJAL–RETZIUS CELL RESEARCH

The first neurochemical characterization of CR cells was performed by Huntley and Jones (1990), who showed that CR cells express calcium-binding proteins such as calbindin and parvalbumin. More recent work has shown that the expression of calcium binding proteins within layer I cell populations is heterogeneous and depends on the species and stage of development (Martinez-Galan et al., 2014). CR cells are glutamatergic (Imamoto et al., 1994; Alcantara et al., 1998; Marin-Padilla, 1998; Meyer et al., 1998; Ina et al., 2007) and best known for their expression of Reln and a series of transcription factors including Tbr1, Tbr2, and P73 (Hevner et al., 2001, 2003; Meyer et al., 2002; Hodge et al., 2013). Reln is synthesized and secreted into the extracellular matrix of the MZ, diffusing through the developing cortex. Reln binds to the ApoR2 and VLDLR receptors expressed by radial glial cells and newborn neurons migrating to the cortical plate (Rice et al., 1998; D’Arcangelo et al., 1999; Rice and Curran, 2001; Tissir and Goffinet, 2003). Reln activates the adapter protein Dad1, and PIK3, leading to serine 3 phosphorylation of cofilin, an actin-depolymerizing protein that promotes the disassembly of F-actin. Phosphorylation at serine 3 renders cofilin unable to depolymerize F-actin, thereby stabilizing the cytoskeleton (Frotscher et al., 2009). CR cells are believed to be crucial for both the development and the evolution of a laminated pattern in the pallium (Trommsdorff et al., 1999; Howell et al., 2000; Kuo et al., 2005; Abellan et al., 2010). In addition, it has been suggested that CR cells signal to ventricular zone progenitors and function as modulators of early cortical patterning (Griveau et al., 2010). For a comprehensive review on the function of Reln protein, see Frotscher et al. (2009).

Recent studies have determined that layer I cells are derived from extracortical sites, including the cortical hem, septum, retrobulbar area, and thalamic eminence (Grove et al., 1998; Meyer and Wahle, 1999; Meyer et al., 1999; Meyer et al., 2002; Abraham et al., 2004; Takiguchi-Hayashi et al., 2004; Bielle et al., 2005; Yoshida et al., 2006; Cabrera-Socorro et al., 2007; Ceci et al., 2010; Gu et al., 2011). The newly generated CR cells migrate tangentially within layer I to reach their destination. Some evidence suggests that the CR cells generated at different sites populate different cortical regions and may thus play distinct, region-specific roles and function in neocortical development (Bielle et al., 2005; Gu et al., 2011). On the other hand, CR cells of different ontogenic origins display comparable functional properties in the early postnatal cortex and may therefore perform similar functions within the transient neuronal networks of the developing cortex (Sava et al., 2010). The distinct origins of CR cells are likely an additional factor that contributes to the heterogeneity of layer I cell types. For more detail on CR cell origins, see Meyer (2010).

Recent studies have also expanded our knowledge of CR cell physiology. CR cells, together with GABAergic interneurons, form a dense network in layer I throughout various neocortical areas, exhibit characteristic membrane patterns and firing patterns, and receive both GABAergic and non-GABAergic input (Anstotz et al., 2013). Modern studies propose, as Cajal did one century earlier, that the source of CR cell inputs include layer I-targeting Martinotti-like interneurons, which express functional group I mGluRs (Cosgrove and Maccaferri, 2012). This suggests that enhanced glutamate release is critical for the establishment of an mGlu1α-dependent micro-circuit, which leads to the activation of CR cells (Cosgrove and Maccaferri, 2012). It has also been shown that GABAergic subplate neurons innervate CR cells. These synaptic connections are thought to be transient and therefore important for neocortical development (Myakhar et al., 2011). For more details, see Luhmann et al. (2014).

DeFelipe together with other 42 experts in cortical cytoarchitechture recently classified cortical interneurons using the “gardener” method for classification and found high consensus for some terms such as Chandellier cell and Martinotti cell. However, this approach found low consensus among experts for the term CR cell (DeFelipe et al., 2013), demonstrating that defining CR cells is not consistent across the field. It is clear that MZ/layer I cells are heterogeneous in morphology, size, location, age, molecular expression, origin, and species. This apparent heterogeneity presents challenges and can potentially seed confusion in reporting results. This is not a new problem as the term “CR cell” has been applied differently for over the last 100 years. For example, CR cells may refer to horizontal cells present only during fetal development (Bradford et al., 1977), or to short axon cells in embryos and adult (Marin-Padilla, 1990), or MZ/layer I cells that express AChE but not GABA (Huntley and Jones, 1990; Soda et al., 2003), or more commonly as the MZ cells that express Reelin (Meyer and Goffinet, 1998). Cell classification can be approached as lumping or splitting. We suggest a definition of CR cells that is more inclusive. In considering the most basic question: what is a Cajal Retzius cell? It is useful to take into account how difficult it is to arrive at an answer:

(1) Cajal, Retzius, Koelliker, Verati, and others disagreed on the identity of CR cells and changed their opinion on this matter.
(2) Cajal first described horizontal cells in layer I of postnatal animals, while Retzius described horizontal cells in human fetuses.
(3) Not all the cells described by Cajal or Retzius were horizontal.
(4) Cajal described multiple morphological categories and subcategories and classifications for cells in layer I (not included in this manuscript).
(5) The cells described by Cajal and Retzius in their original work could be viewed as similar or different, based on morphological criteria.
(6) Original studies used the Golgi method as a principal approach for cell labeling, while current research often uses immunohistochemistry.
(7) Only the Golgi method, rarely used today, labels the entire dendritic arbor of MZ/layer I cells.
(8) The Golgi method may not label all cell types in the MZ or layer I.
(9) Horizontally oriented cells in the MZ of the developing brain will include migrating interneurons originating from the ganglionic eminences, and microglia. Therefore the termination of interneuron migration, and the dispersal of microglia contribute to the decreased cell number in layer I during the postnatal period. These normal developmental processes are distinct from the cell death of CR cells.
(10) Neurons in MZ/layer I originate from a variety of anatomical regions.
(11) The adult cerebral cortex also has Reln-expressing horizontally oriented neurons.
(12) CR cells express pallial and subpallial markers.
(13) Reln is the main protein used to label CR cells. However, Reln does not label all horizontal neurons in layer I, while at the same time labels cells with a morphology matching that of the short axon cells described by Cajal.
(14) The function of adult layer I cells has not been completely determined.
(15) Cajal proposed that both horizontal cells and short axon cells serve similar connectional functions.

To avoid discrepancy due to the heterogeneous nature of cell types in layer I based on measures such as morphology, we propose to use the term “CR cell” to describe a *class* of cells, rather than a single cell type. In the same way that the term “pyramidal neuron” refers to a class of cortical neurons that includes distinct subtypes, we propose to use the term ‘CR cell’ to refer to a class of neurons that includes multiple subtypes based on specific cell morphology, location, age, origin, and marker expression. In this scheme, the term “CR cell” will describe any Reln+ neuron present in the developing MZ and the postnatal/adult layer I of the cerebral cortex. The term “CR cell” will not include the pioneer-neurons of Fairén that emit the earliest descending axonal projection from the cerebral cortex to the subpallium (Morante-Oria et al., 2003), nor will it include migrating interneurons derived from the ganglionic eminences. This approach will create a single class of neurons, CR cells, that comprise multiple heterogeneous subtypes based on molecular and morphological considerations.

## Conflict of Interest Statement

The authors declare that the research was conducted in the absence of any commercial or financial relationships that could be construed as a potential conflict of interest.
